# Projected Northward Expansion and Southern Core-Habitat Contraction of *Zeugodacus tau* in China Under Climate Change: An Optimized MaxEnt Analysis

**DOI:** 10.3390/insects17060596

**Published:** 2026-06-07

**Authors:** Yifu Du, Zhiwen Li

**Affiliations:** College of Plant Protection, Hunan Agricultural University, Changsha 410128, China; duyifu@stu.hunau.edu.cn

**Keywords:** *Zeugodacus tau*, MaxEnt, climate change, climatic suitability, centroid shift, pest risk, species distribution modeling

## Abstract

Global warming is reshaping the climatic suitability of invasive ectotherms. In this study, we used an optimized Maximum Entropy (MaxEnt) model combined with GIS-based centroid tracking to quantify the spatiotemporal changes in the potential climatic suitability of *Zeugodacus tau*, a major polyphagous quarantine pest, in China. Projections were conducted under the historical baseline climate (1970–2000) and future climate scenarios, including SSP1–2.6, SSP2–4.5, and SSP5–8.5 for the 2050s and 2070s. Here, we projected potential climatic suitability rather than future occurrence or abundance. The model showed high predictive performance (AUC = 0.921). Annual mean temperature (Bio1) and mean diurnal range (Bio2) were identified as the primary environmental variables shaping the species’ climatic suitability. Future projections suggested contrasting spatial changes in climatic suitability, with expansion mainly in northern marginal regions and contraction of the southern highly suitable core area. As winter temperatures increased, the low- and moderate-suitability areas were projected to expand northward into temperate agricultural regions. However, under the extreme warming scenario (SSP5–8.5), the highly suitable core area in southern China was projected to decline by 31.61%, while the centroid of the highly suitable area shifted inland and northwestward by approximately 168 km toward the Wuling Mountains. These spatial patterns may be associated with increasing summer heat stress in low-altitude southern regions, although this mechanism requires further physiological validation. Overall, these findings provide a scientific basis for risk-oriented early warning, quarantine planning, and region-specific pest management under future climate change.

## Simple Summary

The South Asian fruit fly (*Zeugodacus tau*) is a highly destructive agricultural pest that causes serious damage to vegetable and fruit crops worldwide. As global temperatures continue to rise, areas with suitable climatic conditions for this pest are expected to shift substantially. In this study, we used spatial modeling approaches to predict changes in the potential climatic suitability of *Z. tau* across China under future climate scenarios. The results suggest a contrasting spatial redistribution of climatic suitability under climate warming: warmer winters may promote northward expansion into temperate agricultural regions, whereas intensified summer heat may be associated with reduced suitability in historically highly suitable southern areas. This pattern is consistent with a possible role of heat-related constraints, but further physiological validation is needed. These findings provide an early-warning reference for agricultural authorities and may help guide risk-oriented monitoring, quarantine planning, and environmentally sustainable pest management in regions that may become climatically suitable.

## 1. Introduction

The South Asian fruit fly, *Zeugodacus tau* (Walker) (Diptera: Tephritidae), is a widely distributed polyphagous quarantine pest. It has a broad host range, with economically important hosts concentrated mainly in Cucurbitaceae and Solanaceae. Females puncture the fruit epidermis to lay eggs, and the subsequent larval feeding causes tissue necrosis, premature fruit drop, secondary pathogen infection, and reduced fruit marketability. Although nationwide quantitative estimates of economic losses caused by *Z. tau* in China remain limited, this pest is recognized as an economically important fruit fly because it damages cucurbitaceous and solanaceous crops and may trigger quarantine or trade restrictions [1,2]. In China, the historical distribution of *Z. tau* has been largely confined to the tropical and subtropical regions of the south and southwest [1]. However, increasing trade and rapid environmental change may elevate its invasion risk and the potential for further spread in China [2,3].

Macroclimatic conditions, particularly temperature, play a central role in determining the distribution, phenology, and survival of ectothermic invasive species [4,5]. Rising mean temperatures are expected to reduce winter cold stress at higher latitudes, thereby weakening historical thermal barriers and facilitating the poleward expansion of tropical pests [4,6]. In contrast, the increasing frequency and intensity of extreme summer heatwaves may impose heat-related constraints on population persistence in historically warm regions [5,7]. Climate warming may therefore generate opposing spatial responses, simultaneously relaxing cold constraints at higher latitudes while increasing heat-related constraints in historically warm regions [4,5,6,7]. Such contrasting responses provide a biological basis for expecting both northward expansion and potential contraction of historically highly suitable southern core areas.

Species distribution models (SDMs) are widely used to forecast pest invasions under climate change [6]. Previous SDM-based studies on fruit flies under climate change have generally emphasized poleward expansion or increases in total suitable area [3,8,9,10,11]. However, less attention has been paid to the possibility that warming may simultaneously reduce climatic suitability in historically highly suitable southern core areas. The interpretation of these spatial projections should also consider the fundamental physiological constraints of the target species [12,13]. According to the Climate Variability Hypothesis (CVH), ectotherms originating from climatically stable, low-latitude environments often possess relatively narrow thermal tolerance breadths and may live close to their upper physiological limits [4,12,13]. Ecophysiological studies further suggest that the critical thermal maximum (CT_max_) of such insects is constrained by the thermodynamic stability of their functional proteins [13]. Accordingly, under extreme greenhouse gas emission scenarios such as SSP5–8.5, historically suitable areas may experience increasing heat stress, potentially favoring persistence in cooler inland or topographically buffered environments [12,14,15].

Because occurrence records of *Z. tau* are concentrated mainly in southern and southwestern China, default MaxEnt settings may increase the risk of overfitting to spatially clustered records. Therefore, model tuning with ENMeval and spatial block cross-validation was used to reduce model complexity and improve transferability under future climate scenarios. In this study, we applied an optimized MaxEnt model combined with spatial centroid tracking to evaluate the spatial redistribution of potential climatic suitability of *Z. tau* in China under future climate change. Specifically, we aimed to quantify changes in climatic suitability under future SSP scenarios, examine whether northern expansion is accompanied by contraction of the southern highly suitable core area, and discuss the biological plausibility of the projected spatial patterns and the major sources of uncertainty [14,15].

## 2. Materials and Methods

### 2.1. Species Occurrence Data and Spatial Thinning

Occurrence records of *Zeugodacus tau* in China were compiled from multiple sources. Initial records were obtained from the Global Biodiversity Information Facility (GBIF) [16]. To ensure broad coverage, additional geographic coordinates were extracted from regional ecological surveys, invasion monitoring studies, and epidemiological reports available through academic databases, including the China National Knowledge Infrastructure (CNKI), VIP, Wanfang, and Web of Science. The dataset was further supplemented with annual plant quarantine bulletins and field monitoring records published by the Ministry of Agriculture and Rural Affairs of China and by provincial plant protection departments. Available temporal metadata were also compiled from the original sources whenever possible, including record years, year intervals, publication years, or other reported temporal information associated with the locality records.

All occurrence coordinates were imported into ArcGIS 10.8 (ESRI, Redlands, CA, USA) for data cleaning. Duplicate records, ambiguous localities, and points located outside terrestrial boundaries were removed. For records without exact collection years, only those with reliable locality information and verifiable sources were retained. Because clustered occurrence records generated by uneven sampling effort can introduce spatial bias and increase the risk of localized overfitting [17,18], spatial thinning was performed using the spThin package version 0.2.0 in R version 4.5.2 (R Foundation for Statistical Computing, Vienna, Austria) [19]. The thinning distance was set to match the spatial resolution of the bioclimatic variables (2.5 arc-minutes, approximately 5 km × 5 km). By retaining only one occurrence point per grid cell, the dataset was reduced to 139 spatially independent records. The 139 final occurrence coordinates were linked to available temporal metadata by exact coordinate matching with the source-year table. The available temporal metadata ranged from 1912 to 2025. Among the final occurrence coordinates, 110 were classified as post-2000 records, 11 as records from 1970 to 2000, 8 as records with temporal intervals crossing the year-2000 boundary, 4 as pre-1970 records, and 6 as records without available year information. The occurrence year was used only to describe the temporal structure of the dataset and was not included as a predictor variable in the MaxEnt model. The final occurrence dataset covered the low-latitude suitable regions of southern and southwestern China and also included records from the Yangtze River Basin, thereby providing a robust basis for ecological modeling. Because the occurrence dataset was compiled from multiple historical and recent sources, some records may not be temporally matched exactly with the 1970–2000 WorldClim baseline climate. This temporal mismatch was considered when interpreting the model projections and is further discussed as a source of uncertainty.

### 2.2. Bioclimatic Variables and Multicollinearity Diagnostics

The predictive performance of ecological niche models depends on both the biological relevance and the statistical independence of the environmental predictors. A total of 19 bioclimatic variables (Bio1–Bio19) were obtained from the WorldClim database (v2.1) at a spatial resolution of 2.5 arc-minutes [20]. Historical climate averages for 1970–2000 were used as the baseline climatic conditions. In this study, this baseline is referred to as the historical baseline climate rather than a true present-day climate. Future climatic suitability projections were generated using the BCC-CSM2-MR global climate model, which was selected because it was developed by the National Climate Center of China and has been widely applied to simulate regional climate patterns in East Asia within the Coupled Model Intercomparison Project Phase 6 (CMIP6) framework [21]. Projections were produced for the 2050s (2041–2060) and 2070s (2061–2080) under three Shared Socioeconomic Pathways: SSP1–2.6, SSP2–4.5, and SSP5–8.5 [22]. SSP1–2.6, SSP2–4.5, and SSP5–8.5 represent low-, intermediate-, and high-emission pathways, respectively.

**Figure A1:**
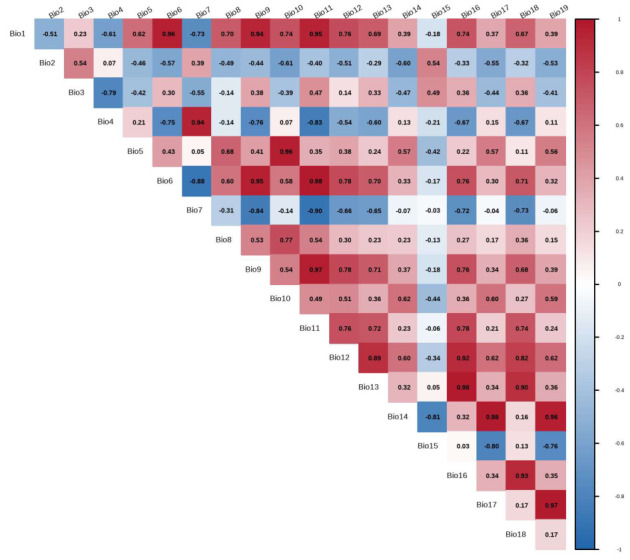
Pearson correlation matrix of the 19 bioclimatic variables. The values within the squares represent the Pearson correlation coefficients (r) between pairs of variables. Positive correlations are indicated in red, while negative correlations are indicated in blue; color intensity is proportional to the correlation coefficient. Variable pairs with an absolute correlation coefficient of |r| ≥ 0.8 were considered to exhibit severe multicollinearity. Based on this matrix, variables with high collinearity were systematically excluded to select the final independent, dominant climatic factors for the ultimate MaxEnt model.

Using all 19 variables in the model can introduce severe multicollinearity, which may inflate parameter variance and reduce the transferability of the model under future climate scenarios [23]. To address this issue, environmental values were extracted for the 139 spatially thinned occurrence records of *Zeugodacus tau*, and Pearson correlation analysis was conducted in R (Figure A1). Variables with an absolute Pearson correlation coefficient of |r| ≥ 0.8 were considered highly collinear. Among correlated variable pairs, retention was determined according to biological relevance and the percent contribution observed during preliminary model runs. This procedure reduced the predictor set to five relatively independent climatic variables: Annual Mean Temperature (Bio1), Mean Diurnal Range (Bio2), Temperature Seasonality (Bio4), Precipitation Seasonality (Bio15), and Precipitation of Coldest Quarter (Bio19). The retained variables and their relative contributions are summarized in Table 1.

### 2.3. Algorithmic Hyperparameter Optimization and Spatial Block Cross-Validation

Ecological niche models based on the Maximum Entropy (MaxEnt) algorithm are sensitive to hyperparameter settings [24,25]. When default settings are used, the model may generate overly complex environmental response curves that fit historical occurrence data too closely, thereby reducing transferability to future climate scenarios [24]. To minimize this risk, we performed parameter optimization in R using the ENMeval package version 2.0.5.2 prior to final model construction [25,26]. The optimization procedure evaluated combinations of Feature Classes (FC), which determine the shape and flexibility of response curves, and the Regularization Multiplier (RM), which penalizes excessive model complexity [24,25]. Six feature-class combinations were tested: Linear (L), Linear-Quadratic (LQ), Hinge (H), Linear-Quadratic-Hinge (LQH), Linear-Quadratic-Hinge-Product (LQHP), and Linear-Quadratic-Hinge-Product-Threshold (LQHPT). These were combined with RM values ranging from 0.5 to 4.0 at intervals of 0.5, yielding 48 candidate models for evaluation.

Because conventional random k-fold cross-validation can inflate model performance when nearby occurrence records are split between training and testing subsets, we used a spatial block cross-validation framework to reduce the influence of spatial autocorrelation [25,27]. The study area in China was partitioned into geographically distinct blocks based on latitude and longitude, and model performance was evaluated by training on a subset of blocks and testing on spatially independent hold-out blocks [25,27]. This procedure provided a more conservative assessment of predictive performance and improved the robustness of model transfer to novel future climates [25].

The optimal parameter combination was selected using information-theoretic criteria [24]. Model selection was based primarily on the corrected Akaike Information Criterion for small sample sizes (ΔAICc), which balances goodness-of-fit against model complexity [24]. We further examined the 10% training omission rate (OR10) and the difference in Area Under the Curve (ΔAUC) to assess prediction failure and overfitting [25]. Based on this multi-criteria evaluation, the optimal model configuration was identified as Linear-Quadratic features (FC = LQ) with a Regularization Multiplier of 1.5 (RM = 1.5), with ΔAICc = 0 and the highest AICc weight among candidate models. This parameterization constrained the model to relatively smooth response curves while reducing the influence of spatial noise and limiting overfitting.

### 2.4. Final Model Construction and Cartographic Projection

The final spatial prediction model was implemented in MaxEnt version 3.4.4 using the optimized parameter settings identified in the tuning procedure (FC = LQ, RM = 1.5) [28]. China’s terrestrial area was used as both the calibration and projection area to assess national-scale climatic suitability and quarantine risk, based on 10,000 background points randomly sampled across the country. All spatially thinned occurrence records were used for final model training, and no additional random test split was applied, because predictive robustness had already been evaluated through spatial block cross-validation in ENMeval [25,26]. Reserving part of the occurrence dataset for a further random split would have unnecessarily reduced the information available for final model fitting and could have weakened extrapolation under future climate scenarios [25]. Subsample replicate runs were used to assess predictive variability without changing the final projection framework. The complementary log-log (cloglog) output format was selected, as it provides suitability estimates on a probability-like scale that is widely used in ecological niche modeling [28]. We acknowledge that the use of a broad national-scale calibration area may influence model discrimination and area estimates; therefore, this issue was considered when interpreting the projected suitable-area changes and is further discussed as a source of uncertainty.

**Figure 1:**
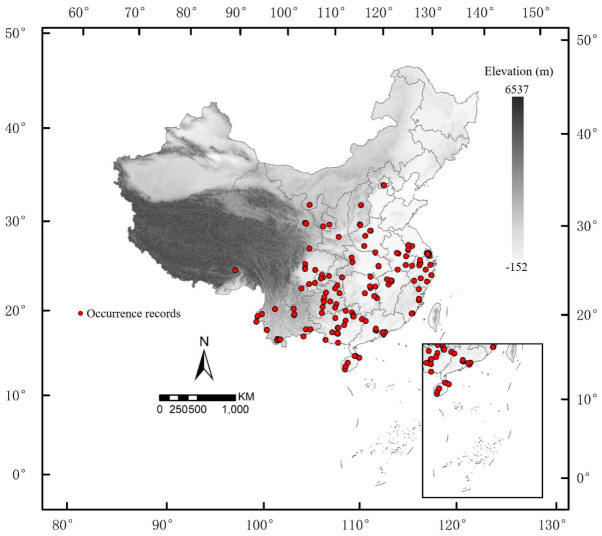
Known geographical distribution of *Zeugodacus tau* in China and the topographic background of the study area. Red dots indicate the 139 occurrence records retained after data cleaning and spatial thinning. The grayscale background represents elevation (m), illustrating the topographic heterogeneity of China. The inset map in the lower right shows the South China Sea Islands and the complete ten-dash line.

Accurate spatial area estimation was required for calculating habitat extent and tracking centroid movement. Because unprojected geographic coordinate systems such as WGS 1984 can introduce areal distortion, especially at higher latitudes, all environmental raster layers and occurrence geometries were transformed to the Asia North Albers Equal Area Conic projection in ArcGIS 10.8 [29,30]. This equal-area projection was used to minimize geometric distortion in subsequent area calculations and centroid extraction [29,30]. For the East Asian domain, the central meridian was set to 105.000000° E, and the standard parallels were set to 25.000000° N and 47.000000° N. The known occurrence records and topographic background of the study area are shown in Figure 1. All maps showing the territory of China in this study were based on the standard map service provided by the Ministry of Natural Resources of the People’s Republic of China (Map Approval Number: GS(2024)0650), and the base map boundaries were not modified.

### 2.5. Ecological Thresholding and Geometric Centroid Tracking

The MaxEnt model generated a continuous cloglog suitability surface ranging from 0 to 1 [28]. To convert this continuous output into ecologically interpretable classes, the minimum training presence (MTP) threshold was first used to distinguish unsuitable from potentially suitable habitats [31,32]. The MTP value was 0.0052, corresponding to the lowest predicted suitability associated with any validated occurrence record in the training dataset. Accordingly, areas with suitability values below 0.0052 were classified as unsuitable habitats.

For all areas with suitability values ≥0.0052, the suitability surface under the historical baseline climate was further classified in ArcGIS 10.8 using the Natural Breaks (Jenks) method to identify internal clusters within the probability distribution [33]. Based on this procedure, the study area was divided into four suitability classes: unsuitable habitats (*p* < 0.0052), low-suitability habitats (0.0052 ≤ *p* < 0.294198), moderate-suitability habitats (0.294198 ≤ *p* < 0.592267), and high-suitability habitats (0.592267 ≤ *p* ≤ 1.0000). To ensure comparability across time periods and climate scenarios, the same classification thresholds derived from the historical baseline climate were consistently applied to all future projections. The MTP/Jenks classification was used for the main four-class suitability framework, whereas the alternative binary thresholds, including the 10th percentile training presence threshold and the equal training sensitivity and specificity threshold, were used only for threshold sensitivity analysis.

To quantify spatial shifts in the species’ highly suitable climatic core under future climate change, we calculated the geometric centroid of the high-suitability habitat class in ArcGIS. Only high-suitability habitats were included in the centroid analysis in order to minimize the influence of marginal suitability zones at the expansion and trailing edges, which are more sensitive to local boundary fluctuations. Centroids were calculated for the high-suitability class only and should be interpreted as the geometric centers of projected climatic suitability rather than population centers or realized distribution centers. This approach allowed us to characterize the direction and magnitude of centroid movement under different future climate scenarios.

### 2.6. Threshold Sensitivity Analysis

To evaluate the robustness of suitable-area estimates to threshold selection, we conducted a threshold sensitivity analysis using two commonly applied binary thresholds derived from the optimized MaxEnt model: the 10th percentile training presence threshold (0.2292, cloglog output) and the equal training sensitivity and specificity threshold (0.3090, cloglog output) [31,32]. The threshold sensitivity analysis was extended from the historical baseline climate to all future projections to evaluate whether projected suitable-area estimates were sensitive to alternative binary threshold choices. Specifically, binary suitable-area estimates were calculated for the historical baseline climate, SSP1–2.6, SSP2–4.5, and SSP5–8.5 in the 2050s and 2070s. For each threshold, the continuous cloglog prediction raster was converted into a binary suitability map in ArcGIS, in which cells with suitability values greater than or equal to the threshold were classified as suitable, whereas cells below the threshold were classified as unsuitable [31,32]. The resulting binary rasters were then projected to the Asia North Albers Equal Area Conic coordinate system using nearest-neighbor resampling and masked to the terrestrial extent of China prior to area calculation. Suitable area was calculated as the number of suitable pixels multiplied by the projected cell area. This supplementary analysis was performed solely to assess the sensitivity of total suitable-area estimates to threshold selection and did not replace the MTP/Jenks four-class suitability framework used in the main analyses. Complete binary suitable-area estimates under the historical baseline and future climate scenarios are provided in Supplementary Table S1.

## 3. Results

### 3.1. Model Accuracy Evaluation and Dominant Environmental Variables

#### 3.1.1. Model Prediction Accuracy Evaluation

**Figure 2:**
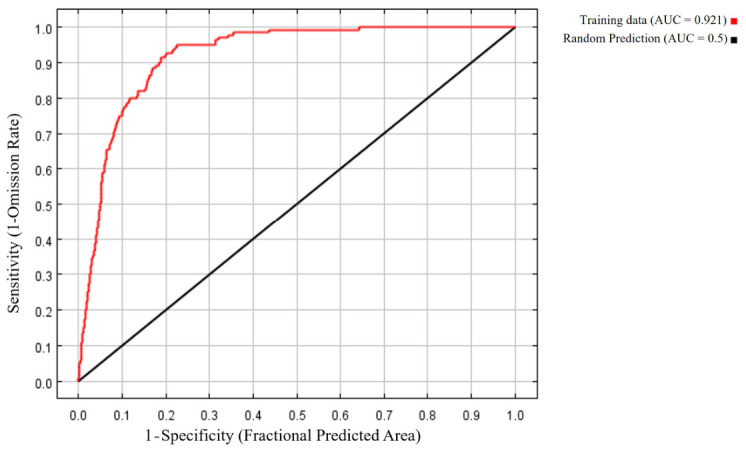
Receiver Operating Characteristic (ROC) curves of the optimized MaxEnt model for *Zeugodacus tau* under spatial block cross-validation. The red solid line represents the mean ROC curve across spatially independent evaluation folds, whereas the black solid line indicates random prediction (AUC = 0.5). The mean validation AUC value of 0.921 indicates strong predictive performance and good model transferability.

Based on 139 spatially independent occurrence records and five selected climatic variables, the optimized MaxEnt model showed strong predictive performance. Parameter tuning in ENMeval identified the optimal model configuration as Linear-Quadratic feature classes (FC = LQ) with a Regularization Multiplier of 1.5 (RM = 1.5). Under this configuration, the model achieved a ΔAICc value of 0 and a mean validation AUC of 0.921. As shown in Figure 2, the optimized model was used for subsequent spatial projections because it balanced model fit and complexity under spatial block cross-validation, thereby reducing the risk of overfitting and improving transferability to future climate scenarios.

#### 3.1.2. Selection and Analysis of Dominant Environmental Variables

**Figure 3:**
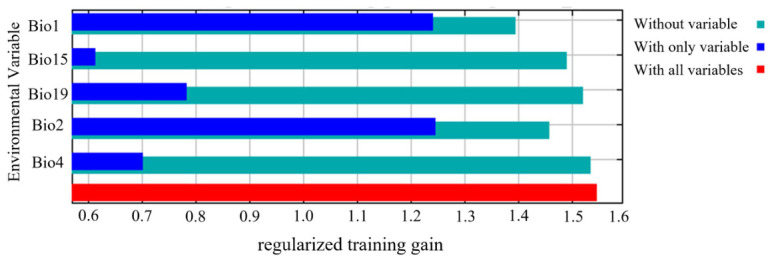
Jackknife test of environmental variable importance for the optimized MaxEnt model of *Zeugodacus tau*. Dark blue bars indicate the regularized training gain when each variable was used in isolation, light blue bars indicate the training gain when each variable was omitted in turn, and the red bar represents the gain of the full model including all variables. A higher gain when a variable is used alone indicates stronger independent predictive power, whereas a greater reduction in gain when a variable is omitted indicates that the variable contributes unique information not contained in the other predictors.

The effects of climatic variables on the potential distribution of *Z. tau* were evaluated using percent contribution, permutation importance, and Jackknife tests. The Jackknife test of regularized training gain revealed clear differences in variable importance depending on the evaluation approach (Figure 3). When used individually, Bio2 produced the highest training gain, followed by Bio1, indicating that Bio2 provided the greatest independent explanatory power for predicting the species’ distribution. In contrast, when variables were omitted one at a time, excluding Bio1 caused the largest reduction in model gain, suggesting that Bio1 contained unique information not captured by the other predictors. Bio19 ranked third in terms of independent predictive contribution and showed a moderate effect on the overall model. Together, these results indicate that temperature-related variables, particularly Bio1 and Bio2, played dominant roles in shaping the potential distribution of *Z. tau*.

### 3.2. Responses of Presence Probability to Dominant Environmental Factors

For visual interpretation of the response curves, predicted probability values above 0.5 were considered to indicate relatively high predicted suitability. This interpretation was independent of the Jenks-based spatial classification used for mapping suitability classes.

**Figure 4:**
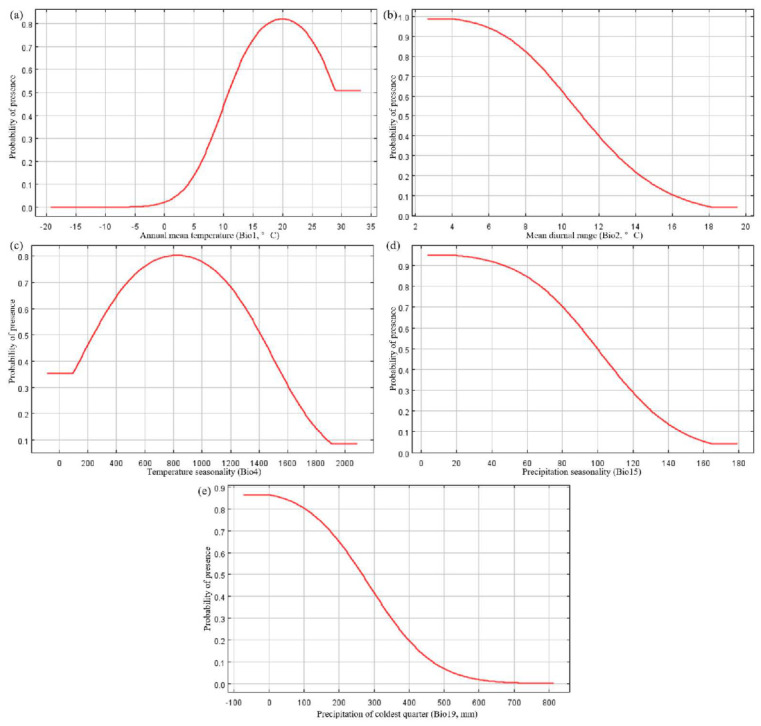
Response curves of the predicted probability of presence of *Zeugodacus tau* in relation to the dominant environmental variables retained in the optimized MaxEnt model. The red solid line represents the predicted response of habitat suitability to each variable. For visual interpretation, predicted probability values greater than 0.5 were considered to indicate relatively high predicted suitability; this interpretation was independent of the Jenks-based spatial classification used for mapping suitability classes. The subplots show the responses to (**a**) Annual mean temperature (Bio1, °C), (**b**) Mean diurnal range (Bio2, °C), (**c**) Temperature seasonality (Bio4), (**d**) Precipitation seasonality (Bio15), and (**e**) Precipitation of coldest quarter (Bio19, mm).

Among the temperature-related variables, the response curve for Annual Mean Temperature (Bio1) showed a unimodal pattern (Figure 4a). The predicted probability of presence for *Z. tau* exceeded 0.5 between 11 °C and 29 °C, with suitability peaking at approximately 20 °C and remaining relatively high at warmer temperatures. For Mean Diurnal Range (Bio2), the predicted probability remained above 0.5 only when the value was below 11 °C, and suitability declined continuously as the daily temperature range increased (Figure 4b). For Temperature Seasonality (Bio4), relatively high predicted suitability occurred approximately between 220 and 1400, with the maximum predicted probability occurring near 800 (Figure 4c).

The two precipitation-related variables, Precipitation Seasonality (Bio15) and Precipitation of the Coldest Quarter (Bio19), both showed negative relationships with habitat suitability. The predicted probability of presence declined as the values of these variables increased. The upper limit of highly suitable conditions (probability > 0.5) was approximately 100 for Bio15 (Figure 4d) and 260 mm for Bio19 (Figure 4e).

### 3.3. Spatial Pattern of Suitable Habitats for Zeugodacus tau in China Under Historical Baseline Climate

**Figure 5:**
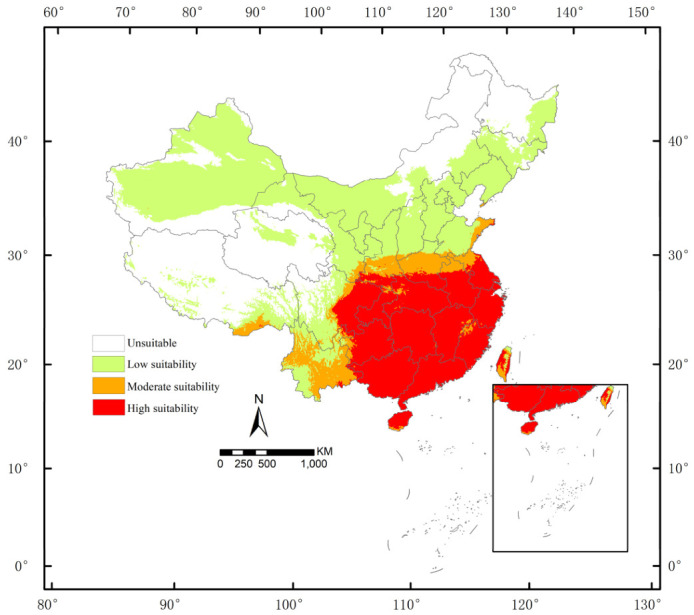
Spatial distribution of potentially suitable habitats for *Zeugodacus tau* in China under the historical baseline climate. White indicates unsuitable habitats, light green indicates low-suitability habitats, orange indicates moderate-suitability habitats, and red indicates high-suitability habitats. The inset map shows the South China Sea Islands and the complete ten-dash line.

The optimized MaxEnt model and pixel-based analyses in ArcGIS revealed the potential geographical distribution and suitable habitat area of *Z. tau* in China under the historical baseline climate (Table 2; Figure 5). Quantitative analysis showed that the total suitable habitat, including low-, moderate-, and high-suitability classes, covered 6.29 × 10^6^ km^2^, accounting for approximately 66.06% of the effective terrestrial study area. The total suitable area estimated under the MTP/Jenks framework included extensive low-suitability habitats and should not be interpreted as the actually occupied range of *Z. tau* or as uniformly high-risk areas. The predicted suitable habitats formed a largely contiguous belt across southern and central-eastern China. Low- and moderate-suitability areas covered 3.73 × 10^6^ km^2^ (39.19%) and 0.63 × 10^6^ km^2^ (6.60%), respectively, and were mainly distributed along the northern margin of the highly suitable zone. These areas showed a clear latitudinal gradient and extended across the Yangtze River Basin to the middle and lower Yangtze River Plain, reaching the southern edge of the Huang-Huai-Hai Plain. In contrast, unsuitable areas covered 3.23 × 10^6^ km^2^ (33.94% of the effective terrestrial study area) and were concentrated primarily in the Qinghai–Tibet Plateau, the arid inland regions of northwestern China, and the cold, high-latitude regions of northeastern China.

Threshold sensitivity analysis further showed that binary suitable-area estimates varied with threshold choice. Across the historical baseline and future climate scenarios, the suitable area estimated using the 10th percentile training presence threshold ranged from 2.62 to 2.82 × 10^6^ km^2^, whereas that estimated using the equal sensitivity–specificity threshold ranged from 2.30 to 2.57 × 10^6^ km^2^. These results indicate that absolute binary suitable-area estimates were threshold-dependent but remained within a comparable range across climate scenarios. Complete threshold sensitivity results are provided in Table S1.

### 3.4. Future Redistribution of Climatic Suitability Under Climate Change

**Figure 6:**
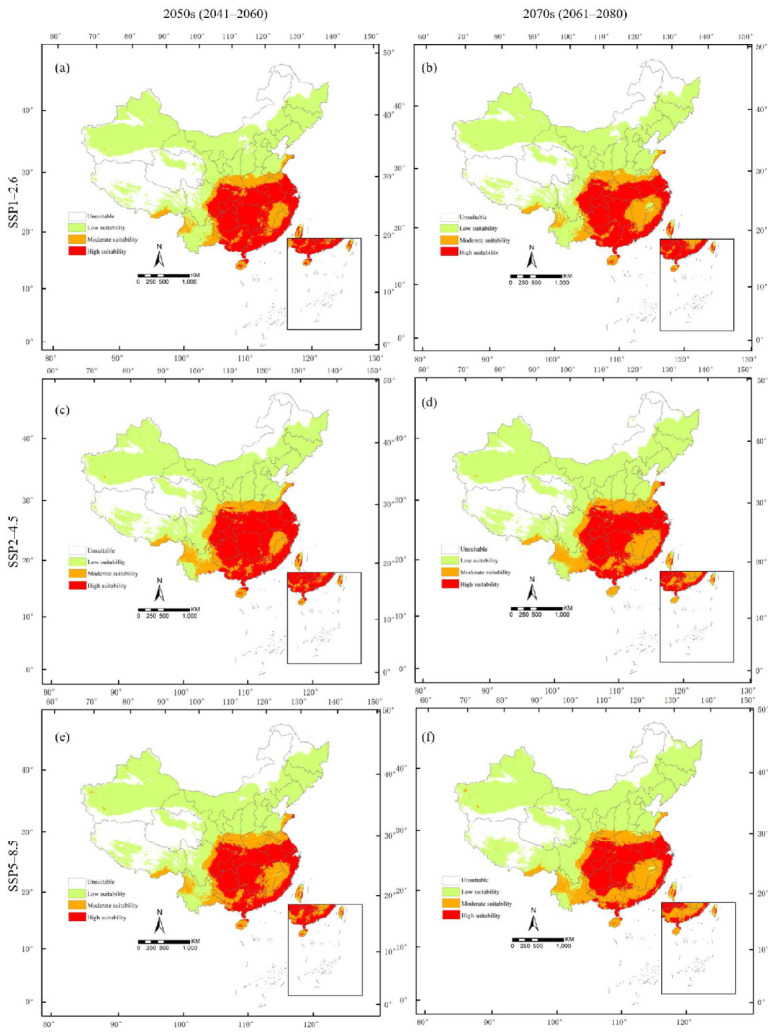
Projected climatic suitability of *Zeugodacus tau* in China under three SSP scenarios in the 2050s and 2070s. Colors indicate the four suitability classes defined in Section 2.5. The same MTP/Jenks classification thresholds derived from the historical baseline climate were applied to all future projections. The inset map shows the South China Sea Islands and the complete ten-dash line. (**a**) SSP1–2.6 in the 2050s; (**b**) SSP1–2.6 in the 2070s; (**c**) SSP2–4.5 in the 2050s; (**d**) SSP2–4.5 in the 2070s; (**e**) SSP5–8.5 in the 2050s; (**f**) SSP5–8.5 in the 2070s.

Under future climate scenarios, both the spatial distribution and the extent of suitable habitats for *Z. tau* were projected to change markedly relative to the historical baseline climate (Figure 6; Table 2). Future projections indicated a redistribution of climatic suitability rather than a uniform expansion of suitable habitats. Expansion mainly occurred in low- and moderate-suitability classes, whereas contraction occurred consistently in the high-suitability core area. Therefore, the projected increase in total suitable area was primarily driven by the expansion of marginal or intermediate suitability zones, rather than by an expansion of the highly suitable core.

Under SSP1–2.6, the high-suitability core area was projected to decrease by 15.54% to 1.63 × 10^6^ km^2^ in the 2050s and by 25.39% to 1.44 × 10^6^ km^2^ in the 2070s. At the same time, low- and moderate-suitability areas expanded toward higher latitudes, partly compensating for the loss of core habitat and resulting in a slight increase in the total suitable area. This pattern suggests a spatial redistribution from high-suitability habitats toward lower-suitability classes under the low-emission scenario.

Under SSP2–4.5, contraction of the high-suitability core area continued, declining to 1.71 × 10^6^ km^2^ in the 2050s and 1.45 × 10^6^ km^2^ in the 2070s. Moderate-suitability areas increased substantially, by 42.86% and 85.71% in the 2050s and 2070s, respectively. Spatially, suitable habitats showed continued northward and inland expansion (Figure 6c,d), whereas the southern core became increasingly fragmented. These changes further indicate that future warming may promote the expansion of marginally and moderately suitable areas while reducing the extent of the highly suitable southern core.

Under the high-emission scenario SSP5–8.5, these shifts were most pronounced. By the 2070s, unsuitable habitat was projected to decrease by 39.94%, whereas low- and moderate-suitability areas increased by 33.78% and 100.00%, respectively, extending further into northern temperate regions. Meanwhile, the high-suitability core area underwent the strongest contraction, decreasing by 31.61% to 1.32 × 10^6^ km^2^. The northern boundary of low- and moderate-suitability habitats continued to expand (Figure 6e,f), whereas historically high-suitability habitats in southern low-latitude regions experienced substantial fragmentation and loss. Thus, under SSP5–8.5, the projected spatial response was characterized by a strong contrast between the northward expansion of lower-suitability classes and contraction of the southern high-suitability core.

### 3.5. Spatial Trajectories of the Centroids of High-Suitability Habitats Under Climate Change

**Figure 7:**
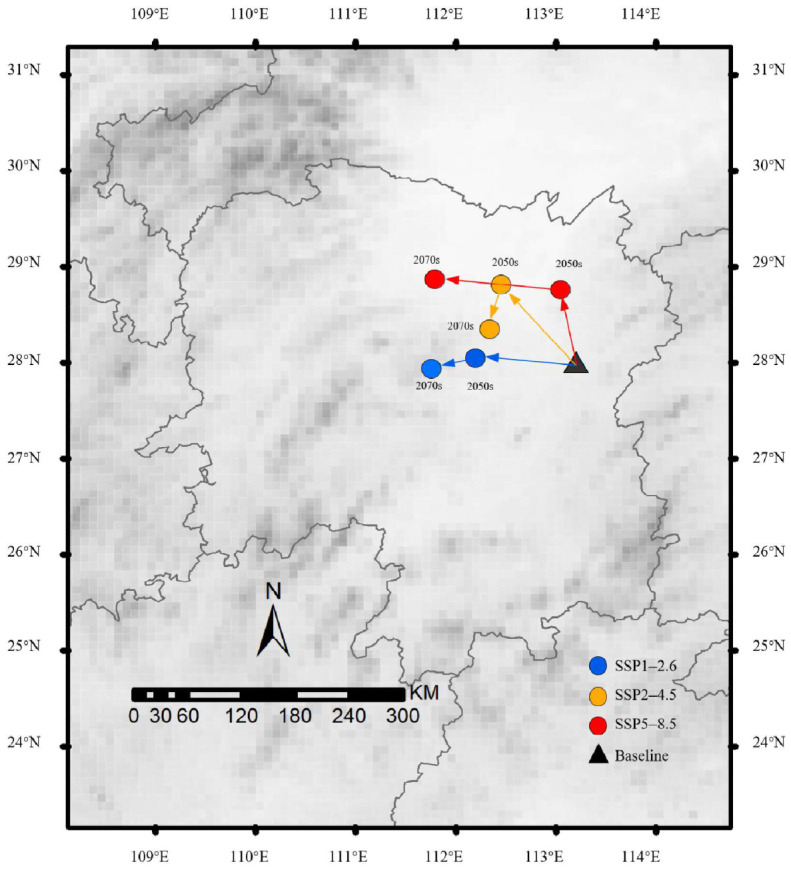
Spatial trajectories of the centroids of high-suitability habitats for *Zeugodacus tau* under different climate change scenarios. The black triangle marks the centroid location under the historical baseline climate. Blue circles and arrows indicate the northwestward shift in the 2050s and southwestward shift in the 2070s under SSP1–2.6, orange circles and arrows indicate the northwestward shifts under SSP2–4.5, and red circles and arrows indicate the northwestward shifts under SSP5–8.5. The gray-shaded background represents the Digital Elevation Model (DEM) of the study area.

The geometric centroids of high-suitability habitats for *Z. tau* were tracked under different climate scenarios to characterize shifts in the species’ climatic core (Figure 7; Table 3). The results revealed clear scenario-dependent migration patterns.

Under the historical baseline climate, the centroid of the high-suitability habitat class was located in central-eastern Hunan Province (113.20° E, 28.00° N). Under SSP1–2.6, the centroid showed a predominantly westward shift. By the 2050s, it had moved approximately 98 km to 112.20° E, 28.05° N, and by the 2070s, it had shifted further westward to 111.76° E, 27.94° N, representing a total displacement of approximately 141 km from the baseline centroid. Under this low-emission scenario, the high-suitability core shifted mainly westward, without evident poleward movement.

Under SSP2–4.5, the centroids followed a northwestward trajectory, indicating inland movement toward higher latitudes. The centroid shifted approximately 93 km northwestward to 112.34° E, 28.35° N in the 2050s and continued to move to 112.46° E, 28.81° N by the 2070s, for a total displacement of approximately 116 km. Under SSP5–8.5, the 2050s centroid first shifted mainly northward, moving approximately 86 km to 113.05° E, 28.76° N. By the 2070s, it had shifted northwestward to 111.79° E, 28.87° N in northwestern Hunan Province, approaching the eastern margin of the Wuling Mountains. The maximum displacement under SSP5–8.5 reached approximately 168 km, indicating a marked inland and poleward shift in the high-suitability climatic core under extreme warming.

## 4. Discussion

### 4.1. Winter Warming and the Potential Northward Expansion of Suitable Habitats

The optimized MaxEnt model showed high predictive performance and stability. Its discriminatory ability is reflected in the AUC results (Figure 2). The model also reproduced the broadly contiguous climatic suitability pattern of *Z. tau* across southern and central-eastern China under the historical baseline climate [1]. Analyses of variable importance indicated that temperature-related variables, especially Annual Mean Temperature (Bio1) and Mean Diurnal Range (Bio2), played dominant roles in shaping the potential distribution of *Z. tau* in China (Figure 3) [15,34]. As an ectotherm, the growth, development, reproduction, and overwintering survival of *Z. tau* are strongly influenced by ambient temperature [1,15,34]. The contrasting response curves observed in this study (Figure 4) are consistent with the thermal performance characteristics typically reported for thermophilic insect pests [14,35].

Historically, low winter temperatures in the Yangtze River Basin may have constrained the northward spread of *Z. tau* into temperate agricultural regions [8,15,34]. Climate warming may alter this pattern. Our projections showed that under both SSP2–4.5 and SSP5–8.5, suitable habitats for *Z. tau* exhibited a general northward expansion trend (Figure 6; Table 2) [8,9,10]. Similar climate-driven range shifts have been reported for other fruit fly pests, including *Anastrepha grandis* and *Drosophila suzukii*, suggesting that climate warming may relax thermal barriers in temperate regions. However, the magnitude and direction of these shifts can differ among species depending on thermal tolerance, host availability, dispersal capacity, and model assumptions [36,37,38]. Although the final predictor set did not directly include minimum winter temperature, the projected northward expansion may be partly consistent with reduced cold constraints under climate warming [15,34,39]. As winter temperatures rise, overwintering survival of vulnerable life stages, particularly soil-dwelling pupae, may improve, thereby increasing the probability of successful establishment in the following growing season [15,40,41].

This projected northward expansion is unlikely to be driven by temperature alone [39,42]. Temperature Seasonality (Bio4) and Precipitation of the Coldest Quarter (Bio19) also contributed to the modeled distribution [40,42]. The response curves suggest that *Z. tau* is sensitive to strong seasonal thermal fluctuations and to excessive winter precipitation [15,40,43]. Changes in thermal seasonality and winter precipitation may therefore also influence habitat suitability in marginal northern ecotones [8,40]. Taken together, these changes suggest that warming may be associated with a weakening of historical overwintering constraints and an increased likelihood of *Z. tau* establishment beyond the Yangtze River Basin, including parts of the Huang-Huai-Hai Plain [2,8,39].

### 4.2. Potential Role of Summer Heat Stress in the Contraction of Southern Core Habitats

Moderate warming may facilitate spatial expansion, whereas extreme warming can impose contrasting constraints on insect populations [13,44]. In the present study, the model projected a contraction of high-suitability habitats in southern China under extreme warming. Under the high-emission scenario SSP5–8.5, the high-suitability core area of *Z. tau* was projected to decline markedly by the 2070s (Table 2). The projected contraction of the southern high-suitability core area is the most distinctive result of this study, but it should be interpreted as a hypothesis-generating pattern rather than direct evidence of heat-stress-driven decline. Centroid tracking further suggested a northwestward shift from the low-altitude plains of eastern Hunan toward the eastern foothills of the Wuling Mountains (Table 3; Figure 7). The northwestward shift in the high-suitability core area toward the Wuling Mountains may indicate the potential importance of topographic heterogeneity and thermal refugia under extreme warming. When interpreted together with the DEM background, this pattern is consistent with a tendency for the species’ climatic core to move toward cooler inland or higher-elevation environments under intensified warming [45,46,47].

This projected pattern may be associated with increasing summer heat stress in low-altitude southern regions [13,14,15]. Published studies on *Z. tau* indicate limited tolerance to extremely high temperatures [14]. More broadly, studies of tropical and low-altitude insects suggest that upper thermal limits may be constrained by protein thermodynamic stability and limited adaptive plasticity [13,48,49]. Under such conditions, populations in historically suitable southern core habitats may become increasingly exposed to severe summer heat stress, which could contribute to declining habitat suitability [13,44]. However, because the final predictor set did not include explicit indices of summer heat extremes, this interpretation should be regarded as a biologically plausible hypothesis rather than a directly tested mechanism.

The projected centroid shift toward the Wuling Mountains, where elevation exceeds 1200 m in many areas, further suggests a movement toward environments with greater topographic complexity and stronger microclimatic buffering [45,46]. Such montane habitats may provide cooler microhabitats that partially alleviate thermal stress [46,47]. This pattern supports the possibility that topographically heterogeneous landscapes may function as thermal refugia for high-suitability climatic conditions under extreme warming. Nevertheless, this interpretation should be treated with caution. Because the present study did not explicitly incorporate physiological thresholds as predictor variables or apply post hoc thermal masks, the role of thermal tolerance should be regarded as a biologically plausible explanatory hypothesis rather than a directly tested mechanism.

Earlier distributional studies of tephritid pests often relied on older climate datasets or on standard correlative models that emphasized broad poleward or range expansion under warming [3,8,9]. By using CMIP6 climate scenarios and an optimized MaxEnt framework, our results suggest a more complex spatial redistribution of climatic suitability, characterized by simultaneous northward expansion of the total suitable area and contraction of the southern high-suitability core area [3,8,9]. Although the underlying role of heat stress requires explicit physiological testing in future work, the present analysis provides a more nuanced basis for agricultural quarantine planning and climate-adaptive pest management.

### 4.3. Future Risk-Oriented Areas and Implications for Integrated Pest Management

Climate warming may promote the expansion of *Zeugodacus tau* into temperate agricultural regions [1,2,11]. In newly suitable frontier zones, such as the middle and lower reaches of the Yangtze River Basin, higher ambient temperatures may accelerate the overall development rate of the pest and potentially increase the number of generations per year [1,11,34]. Such demographic changes could elevate local population pressure and increase the likelihood of more frequent pesticide applications [39,50]. In this context, establishing robust early-warning and monitoring networks in northern frontier regions will be critical for tracking population dynamics and reducing the risk of large-scale outbreaks [1,11,39]. In newly suitable frontier zones, especially parts of the Yangtze River Basin, monitoring could prioritize cucurbit crops such as cucumber, melon, pumpkin, loofah, and wax gourd, as well as solanaceous crops such as eggplant and tomato.

In contrast, management priorities may differ in historically persistent southern areas, where extreme summer heat is projected to reduce habitat suitability in low-altitude regions [13,14]. In these areas, surveillance efforts should focus more closely on mountainous or topographically buffered habitats where residual populations may persist [45,46,47]. Such locations could serve as local sources for re-establishment once thermal stress is temporarily alleviated [45,47].

Taken together, these results suggest that future Integrated Pest Management (IPM) strategies for *Z. tau* should be region-specific and climate-informed [1,39,50]. In expanding northern risk zones, monitoring, rapid response, and quarantine enforcement should be strengthened to prevent establishment and spread [2,11,39]. In southern persistent habitats, management should prioritize targeted surveillance and non-chemical suppression [1,50]. Continuous reliance on single-target chemical control should be avoided whenever possible [1]. Instead, environmentally sustainable approaches, including lure-based male trapping or attract-and-kill systems, together with the conservation or augmentation of natural enemies, may provide more durable control [1,51,52]. In addition, strict quarantine measures remain essential for limiting human-mediated dispersal, and validated cold-disinfestation schedules during transport remain important for reducing the risk of long-distance movement [1,41].

### 4.4. Model Uncertainties and Future Perspectives

The predictive framework of this study is based on a correlative species distribution modeling approach and therefore relies on the assumption of niche conservatism [53]. MaxEnt also assumes that occurrence records approximately reflect suitable environmental conditions, but *Zeugodacus tau* is an expanding pest and may not yet occupy all climatically suitable areas in China [28,54]. In other words, the climatic associations inferred from the compiled occurrence records of *Z. tau* are assumed to remain broadly informative when projected to future climate scenarios [53]. Another source of uncertainty is the temporal heterogeneity of the occurrence dataset. Some occurrence records were compiled from sources after 2000, whereas the baseline climate represented 1970–2000 averages. This mismatch may introduce uncertainty, particularly because the distribution of this pest in China may have changed in recent decades. Therefore, the model outputs should be interpreted as broad-scale potential climatic suitability rather than exact present-day occurrence or actual population abundance [54,55].

Several methodological choices may also influence the projected suitable-area estimates. The use of the terrestrial extent of China as the calibration area was appropriate for national-scale quarantine risk assessment, but a broad calibration area may influence model discrimination and area estimates [17,18]. In addition, area estimates were sensitive to threshold selection, particularly for the total suitable area [31,32]. Therefore, absolute area values should be interpreted more cautiously than relative patterns among scenarios, such as the contrast between the expansion of low- and moderate-suitability areas and the contraction of the southern high-suitability core area. The model also did not include potentially important non-climatic factors such as host plant distribution, land-use change, dispersal barriers, or human-assisted spread, all of which may influence the realized distribution of this pest [42,54]. Moreover, the present study did not explicitly incorporate physiological thermal thresholds into the modeling process [56]. Therefore, the potential role of heat stress should be regarded as a biologically plausible explanatory hypothesis rather than a directly tested mechanism [53,56].

In addition, future projections were based on a single CMIP6 global climate model (BCC-CSM2-MR) [21]. Although BCC-CSM2-MR is regionally relevant for East Asia and China, the use of a single GCM cannot capture inter-model variability among climate projections [21,55,57]. Consequently, the magnitude of projected range expansion or contraction should be interpreted as scenario-dependent rather than deterministic [55,57]. The model also assumes relatively stable climatic niches. However, rapid evolutionary adaptation or transgenerational plasticity may alter heat tolerance and could partly mitigate the projected contraction of high-suitability areas in southern regions [58]. Future studies should incorporate multiple GCMs, host availability, land-use dynamics, and dispersal processes to improve projection robustness [42,54,55]. It would also be valuable to develop physiology-informed or eco-evolutionary species distribution models that explicitly integrate thermal tolerance data, population-level adaptive potential, and rapid evolutionary responses under extreme warming [53,56,58,59]. Such efforts would help refine invasion risk assessment for emerging agricultural regions under climate change [3,42].

## 5. Conclusions

This study evaluated the effects of climate change on the potential climatic suitability of *Zeugodacus tau* in China. The results indicate that climate warming may substantially alter the projected spatial pattern of suitable habitats for this pest. These findings should be interpreted as projections of potential climatic suitability rather than direct forecasts of future occurrence or abundance. Increasing temperatures may relax cold-related constraints in northern temperate agricultural regions, thereby contributing to the projected expansion of low- and moderate-suitability areas. Under extreme warming scenarios, the model projected a marked contraction of the southern high-suitability core area, accompanied by a northwestward shift in the spatial centroid toward the Wuling Mountains. The contraction of the southern high-suitability core area may be associated with heat-related constraints, although this hypothesis requires further physiological validation.

Taken together, these findings suggest a contrasting spatial redistribution of climatic suitability for *Z. tau* under future climate change, characterized by the expansion of lower-suitability classes in northern frontier regions and contraction of the southern high-suitability core area. The projections provide an early-warning reference for climate-informed surveillance and quarantine planning. In newly suitable frontier zones, monitoring and rapid response may help detect potential establishment at an early stage, whereas in historically suitable southern regions, surveillance may need to consider topographically buffered habitats where suitable climatic conditions could persist. Because the model was based on climatic suitability rather than realized population dynamics, these management implications should be regarded as risk-oriented guidance rather than fixed control recommendations.

## Figures and Tables

**Table 1:** Dominant environmental variables retained for the final MaxEnt model and their relative contributions.

Variable	Description	Percent Contribution (%)	Permutation Importance (%)
Bio1	Annual Mean Temperature (°C)	17.2	69.8
Bio2	Mean Diurnal Range (°C)	73.6	12.3
Bio4	Temperature Seasonality (Coefficient of Variation)	0.8	4.4
Bio15	Precipitation Seasonality (Coefficient of Variation)	6.3	11.8
Bio19	Precipitation of Coldest Quarter (mm)	2.2	1.6

**Table 2:** Predicted suitable habitat areas of *Zeugodacus tau* under the historical baseline and future climate scenarios in China.

Scenario	Period	Unsuitable Habitats		Low-Suitability Habitats		Moderate-Suitability Habitats		High-Suitability Habitats	
		Area (10^6^ km^2^)	Area Change (%)	Area (10^6^ km^2^)	Area Change (%)	Area (10^6^ km^2^)	Area Change (%)	Area (10^6^ km^2^)	Area Change (%)
Historical baseline	Baseline	3.23	-	3.73	-	0.63	-	1.93	-
SSP1–2.6	2050s	2.75	−14.86	4.33	16.09	0.8	26.98	1.63	−15.54
	2070s	2.75	−14.86	4.41	18.23	0.92	46.03	1.44	−25.39
SSP2–4.5	2050s	2.41	−25.39	4.5	20.64	0.9	42.86	1.71	−11.4
	2070s	2.35	−27.24	4.55	21.98	1.17	85.71	1.45	−24.87
SSP5–8.5	2050s	2.48	−23.22	4.53	21.45	1.04	65.08	1.46	−24.35
	2070s	1.94	−39.94	4.99	33.78	1.26	100	1.32	−31.61

**Table note:** Areas were estimated using the MTP/Jenks four-class suitability framework. Area change (%) was calculated relative to the historical baseline. Suitable habitats indicate potential climatic suitability rather than realized distribution or uniformly high-risk areas.

**Table 3:** Geographic coordinates and migration characteristics of the centroids of highly suitable habitats for *Zeugodacus tau* under different climate scenarios.

Period	Climate Scenario	Longitude (°E)	Latitude (°N)	Approximate Shift Distance (km)	Approximate Shift Direction
Historical baseline	Baseline	113.20	28.00	-	-
2050s	SSP1–2.6	112.20	28.05	98	Northwestward (NW)
	SSP2–4.5	112.34	28.35	93	Northwestward (NW)
	SSP5–8.5	113.05	28.76	86	Northwestward (NW)
2070s	SSP1–2.6	111.76	27.94	141	Southwestward (SW)
	SSP2–4.5	112.46	28.81	116	Northwestward (NW)
	SSP5–8.5	111.79	28.87	168	Northwestward (NW)

**Table note:** Centroids were calculated for the high-suitability habitat class using the MTP/Jenks classification framework. Shift distances and directions were calculated relative to the centroid under the historical baseline climate. Shift distances are presented as approximate values rounded to the nearest kilometre to avoid implying excessive precision. These centroids represent geometric centers of projected climatic suitability and should not be interpreted as realized distribution centers or population centers of *Z. tau*.

## Data Availability

Occurrence records were compiled from GBIF occurrence download (DOI: 10.15468/dl.sh7vmu), published literature, and official plant quarantine reports, as described in the Materials and Methods. The cleaned occurrence dataset, suitability-threshold settings, and SDM parameter configurations supporting the findings of this study are openly available in Zenodo at https://doi.org/10.5281/zenodo.19597050 (accessed on 3 June 2026). The baseline and future bioclimatic variables used for modeling were obtained from the WorldClim database (v2.1).

## Supplementary Materials

The following supporting information can be downloaded at: https://www.mdpi.com/article/10.3390/insects17060596/s1, Table S1: Binary suitable-area estimates for *Zeugodacus tau* under historical baseline and future climate scenarios using alternative suitability thresholds.

## Abbreviations

The following abbreviations are used in this manuscript:

MaxEnt	Maximum Entropy
SDMs	Species Distribution Models
CMIP6	Coupled Model Intercomparison Project Phase 6
SSPs	Shared Socioeconomic Pathways
ROC	Receiver Operating Characteristic
AUC	Area Under the Curve
MTP	Minimum Training Presence
10PTP	10th Percentile Training Presence
FC	Feature Class
RM	Regularization Multiplier
DEM	Digital Elevation Model
IPM	Integrated Pest Management
