# Supplementary material for: Projected Northward Expansion and Southern Core-Habitat Contraction of Zeugodacus tau in China Under Climate Change: An Optimized MaxEnt Analysis
Source: Insects. 2026 Jun 7;17(6):596. doi: 10.3390/insects17060596 (PMC13299406; doi:10.3390/insects17060596)
Supplement: Supplementary file 1 [file insects-17-00596-s001.zip › insects-4294780-supplementary.pdf]

Supplementary Materials

Table S1. Threshold sensitivity analysis of binary suitable habitat area for *Zeugodacus tau* under the historical baseline climate and future climate scenarios.

Scenario	Period	10th percentile training presence (0.2292)			Equal sensitivity–specificity (0.3090)		
		Suitable pixels (n)	Suitable area (10^6 km^2)	Suitable proportion(%)	Suitable pixels (n)	Suitable area (10^6 km^2)	Suitable proportion(%)
Historica l baseline	Baseline	119,819	2.74	28.83	109,626	2.51	26.38
SSP1-2.6	2050s	116,842	2.67	28.12	104,478	2.39	25.14
SSP1-2.6	2070s	114,585	2.62	27.57	100,669	2.30	24.22
SSP2-4.5	2050s	122,754	2.81	29.54	111,724	2.56	26.88
SSP2-4.5	2070s	122,362	2.80	29.44	112,434	2.57	27.05
SSP5-8.5	2050s	120,781	2.76	29.06	106,908	2.45	25.73
SSP5-8.5	2070s	123,393	2.82	29.69	110,473	2.53	26.58

Table note: Binary suitable-area estimates were derived from the continuous cloglog output of the optimized MaxEnt model under the historical baseline climate and future climate scenarios. For each climate scenario, cells with predicted suitability values greater than or equal to the corresponding binary threshold were classified as suitable, whereas cells below the threshold were classified as unsuitable. Binary rasters were projected to the Asia North Albers Equal Area Conic coordinate system using nearest-neighbor resampling and clipped to the terrestrial extent of China. Suitable area was calculated as the number of suitable pixels multiplied by the projected cell area of 22.8886 km^2 per cell.
